# Supplementary material for: Mechanically flexible mid-wave infrared imagers using black phosphorus ink films
Source: Nat Commun. 2025 Jul 1;16:5972. doi: 10.1038/s41467-025-60942-8 (PMC12215355; doi:10.1038/s41467-025-60942-8)
Supplement: Supplementary file 1 — Supplementary Information [file 41467_2025_60942_MOESM1_ESM.pdf]

SUPPLEMENTARY INFORMATION

Mechanically flexible mid-wave infrared imagers using black phosphorus ink films

Theodorus Jonathan Wijaya[1,2,3,4,†], *Naoki Higashitarumizu*[1,2,3,†], *Shifan Wang*[1,2,3,5], *Shogo Tajima*[1,3,6], *Hyong Min Kim*[1,2,3], *Shu Wang*[2,3,7], *Dehui Zhang*[1,2,3], *James Bullock*[5], *Tomoyuki Yokota*[4,*], *Takao Someya*[4,*], *and Ali Javey*[1,2,3,7,8,*]

[1]Department of Electrical Engineering and Computer Sciences, University of California, Berkeley, California 94720, U.S.A.

[2]Materials Sciences Division, Lawrence Berkeley National Laboratory, Berkeley, California 94720, U.S.A.

[3]Berkeley Sensor & Actuator Center, University of California, Berkeley, California 94720, U.S.A.

[4]Department of Electrical Engineering and Information Systems, The University of Tokyo, 7-3-1 Hongo, Bunkyo-ku, Tokyo 113-8656, Japan

[5]Department of Electrical and Electronic Engineering, University of Melbourne, Melbourne, Victoria 3010, Australia

[6]Azbil North America Research and Development, Inc., Santa Clara, California 95051, U.S.A

[7]Department of Materials Science and Engineering, University of California, Berkeley, California 94720, U.S.A.

[8]Kavli Energy NanoScience Institute at the University of California, Berkeley, California 94720, U.S.A.

[†]These authors contributed equally.

[*]Corresponding authors

E-mail: ajavey@berkeley.edu, someya@ee.t.u-tokyo.ac.jp, yokota@ntech.t.u-tokyo.ac.jp

Contents

Supplementary Note 1. Ink refinement 3
Supplementary Note 2. Flexibility test of BP film 3
Supplementary Note 3. Analysis of noise characterization and specific detectivity D^* 3

Supplementary Table 1. Properties of BP inks explored in this study (from 300 nanosheets) 4
Supplementary Table 2. Photodetectors realized through liquid-phase exfoliated BP ink 5
Supplementary Table 3. Specifications of MWIR imagers compared to those in this study 6

Supplementary Figure 1. Energy level alignment plot of the photodetector … 8
Supplementary Figure 2. Coating of BP ink film on 10 cm-diameter plastic film 9
Supplementary Figure 3. Raman spectrum of the BP ink 10
Supplementary Figure 4. MWIR photoresponse of the BP PD under λ = 3.3 μm LED… 11
Supplementary Figure 5. Noise characterization and specific detectivity D^* analysis 12
Supplementary Figure 6. Transient rise and fall behavior of the unbiased BP photodetector 13
Supplementary Figure 7. Repeated switching cycles of an unbiased BP photodetector 14
Supplementary Figure 8. Design of the imager 15
Supplementary Figure 9. Variation analysis of responsivity of the imager 16
Supplementary Figure 10. Imaging of the letters "C", "A", and "L" 17
Supplementary Figure 11. Imaging using λ = 3.3-μm LED (33 mW·cm^{-2}) 18
Supplementary Figure 12. Responsivity during the operational stability test… 19
Supplementary Figure 13. Bending test of the BP photodetectors fabricated on PI films 20
Supplementary Figure 14. Bending test of BP film coated on PI (125 μm)/PEDOT:PSS 21
Supplementary Figure 15. Spatial mapping of photocurrent from 1×8 flexible imager… 22
Supplementary Figure 16. Schematic illustration of the applicator 23

References 23

Supplementary Note 1. Ink refinement.

Four ink recipes (A–D) were studied in this work, with variations in sonication time and centrifugation. Among them, MWIR (up to 4 μm) was detected only with devices made using Ink B and Ink C. Ink C outperformed Ink B in terms of the photocurrent (I_{ph}) of the photodetectors at 0 V. The maximum photocurrent (I_{ph}) of photodetectors made using Ink B was ~6 nA, whereas that of photodetectors made using Ink C was ~184 nA. Additionally, Ink C resulted in 100% yield rate of MWIR detection at 0 V, while Ink B resulted in less than 100% yield. As a result, Ink C was selected as the recipe for the photodetectors.

Supplementary Note 2. Flexibility test of BP film.

The conductivity of the BP films were characterized under various bending curvatures by measuring the current through the film with a constant 1 V bias. A significant decrease in measured current was observed, attributed to contact degradation and detachment during bending, which is extrinsic to the conductivity of the BP film and flexibility. To eliminate these effects, we used a graphite-doped paste as a conductive adhesive between the BP film and Al foil contacts[1]. The paste forms a strong adhesion with both the BP film and the metal contacts, minimizing interface damage. Additionally, with a zero Young's modulus due to its viscous liquid nature, the paste relieves excess strain between the rigid contact and the flexible film. The graphite paste was prepared by dispersing powdered graphite in Coke (Coca-Cola, original flavor) at 0.1 $g \cdot mL^{-1}$, then heating and concentrating it until it became viscous. The resistivity of one syrup drop was measured to be below 1 kΩ, which is orders of magnitude lower than the channel conductivity of the BP film.

Supplementary Note 3. Analysis of noise and specific detectivity D^*.

The unbiased device exhibited a noise spectral density (NSD) of 2.3×10^{-13} $A\cdot Hz^{-1/2}$ at 10 Hz (Supplementary Figure 5a) and a corresponding noise equivalent power (NEP) of 1.2×10^{-8} $W\cdot Hz^{-1/2}$ at 3 μm (Supplementary Figure 5b), calculated using NEP = NSD/R, where R is the responsivity at the specified wavelength. The specific detectivity (D^*) was determined using $D^* = \sqrt{A\Delta f}/\mathrm{NEP}$, where A is the device area and Δf is the integration time (1 s), yielding a detectivity of 4.3×10^{6} Jones at $\lambda = 3$ μm. Supplementary Figure 5c shows the variation of D^* with photon energy. Further enhancement of responsivity is required to improve detectivity. To contextualize our device performance, Supplementary Table 3 compares the D^* of our ink-based BP photodetector to that of other reported devices.

Supplementary Table 1. Properties of BP inks explored in this study (from 300 nanosheets).

Ink sample	Sonication time (min)	Centrifugation	Thickness (nm)	Lateral size (nm)	Aspect ratio	MWIR detection	Note
A	10	4014 *g*, 30 min	182 ± 114	756 ± 358	4.1 ± 3.3	- Undetected	
B	30	4014 *g*, 30 min	47 ± 29	351 ± 249	7.5 ± 7.1	✓ Low I_{ph}	
C (Main article)	60	250 *g*, 10 min → 4014 *g*, 30 min	24 ± 15	416 ± 124	17 ± 12	✓ High I_{ph}	Yield ~100%
D	180	250 *g*, 10 min → 4014 *g*, 30 min	12.9 ± 8.6	346 ± 94	27 ± 3.3	- Undetected	

Supplementary Table 2. Photodetectors realized through liquid-phase exfoliated BP ink.

Ref.	Method	Mean BP thickness (nm)	MWIR detection, Maximum detected λ	Imager	Detection at 0 V	Demonstrated flexibility
This work	Refined probe sonication and centrifugation steps	24 ± 15	✓ Mid-wave IR, 4 µm	✓	✓ (Confirmed photodiode behavior)	✓
N. Gupta, *et al.*, (2023)[2].	Probe sonication	47 ± 29	✓ Mid-wave IR, 4 µm	– Single pixel	– (Reverse bias operation)	–
A. Corletto *et al.*, (2024)[3].	Electrochemical exfoliation	6.2	– Short-wave IR, 2 µm	✓	× (Photoconductor)	–
S. Akhavan *et al.*, (2023)[4].	Bath sonication	6.7	× Short-wave IR, 2.7 µm	– Single pixel	× (Photoconductor)	–
G. Hu *et al.*, (2017)[5].	Bath sonication	3.4	– Short-wave IR, 1.55 µm	– Single pixel	–	–

Explanatory Note: The table presents only the reported data, with unreported features indicated by '–'. Some studies explicitly identify the photoconductive mechanism for photodetection, excluding operation at 0 V, while others do not mention operation at 0 V.

Supplementary Table 3. Specifications of MWIR imagers compared to those in this study.

Ref.	Material	MWIR range (μm)	Flexi-bility	Unbiased operation	Uncooled operation	*D** (Jones)	Rise time	Note
This study	BP	3–4	✓	✓	✓	4.3×10^{6} (λ = 3.0 μm)	4 μs	Demonstra-ted scalability
Other imager reports								
G. Mu *et al.*, (2024)[6].	PbS/HgTe	3–5	–	×	✓	3.9×10^{9} (1500 °C source)	350 μs	Toxic compound (Hg, Pb)
T. Xu *et al.*, (2024)[7].	BP/MoS_2	3–4	–	✓	✓	1.1×10^{9} (λ = 3.0 μm)	88 μs	Limited scalability (manual exfoliation)
Commercial imager products								
FLIR A6780	InSb	3–5	×	×	×			Thermal camera
Hamamatsu P5968-060	InSb	3–5	×	✓	(77 K operation)	1×10^{11} (λ_{peak} = 3.0 μm, 77 K) 1.6×10^{11} (λ_{peak} = 5.3 μm, 77 K)	30 ns	
Hamamatsu P16702-011MN	InAsSb	3–8	×	✓	✓	1.6×10^{6} (λ = 3.0 μm) 8.8×10^{6} (λ_{peak} = 7.4 μm)	4 ns	Toxic compound (As)
Thorlabs VML8T0	HgCdTe	3–8	×	✓	✓	3.5×10^{8} (λ = 3.0 μm) 6.0×10^{6} (λ_{peak} = 6.3	4 ns	Toxic compound (Hg, Cd)

						μm)		
Hamamatsu P16309-01	(QCIP)	~3.5–6	×	✓	✓	1.5×10^9 (λ_{peak} = 4.65 μm)	2 ps	

Explanatory note: The table presents only the reported data, with unreported features indicated by '–'. The MWIR range shows the intersection between reported data and the whole MWIR range (λ = 3–8 μm). Some studies explicitly identify the photoconductive mechanism for photodetection, excluding operation at 0 V, while others do not mention operation at 0 V. Some detectivity values for commercial products were calculated from specification sheets, using data at their peak wavelength and at λ = 3.0 μm at room temperature, unless otherwise noted. The rise time of commercial products is estimated by their RC-limited response, $t_{rise} \approx 0.35/f_{3dB}$, where f_{3dB} is the reported cutoff frequency. QCIP: quantum cascading infrared photodetectors.

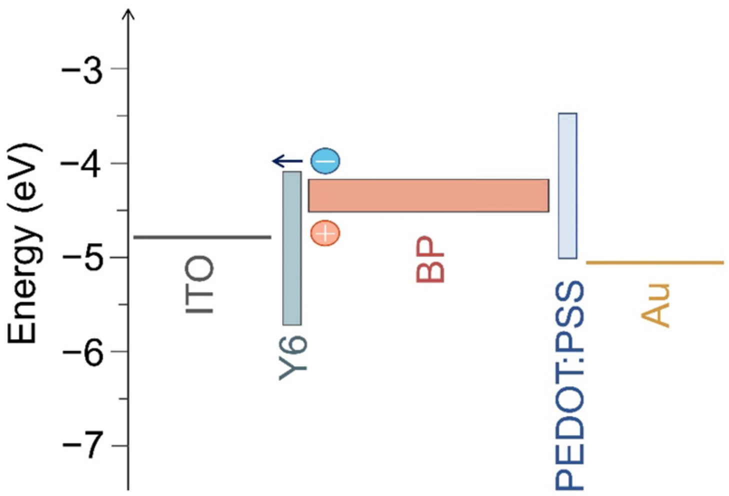

Supplementary Figure 1. Energy level alignment plot of the photodetector under flat band condition between the two electrodes.

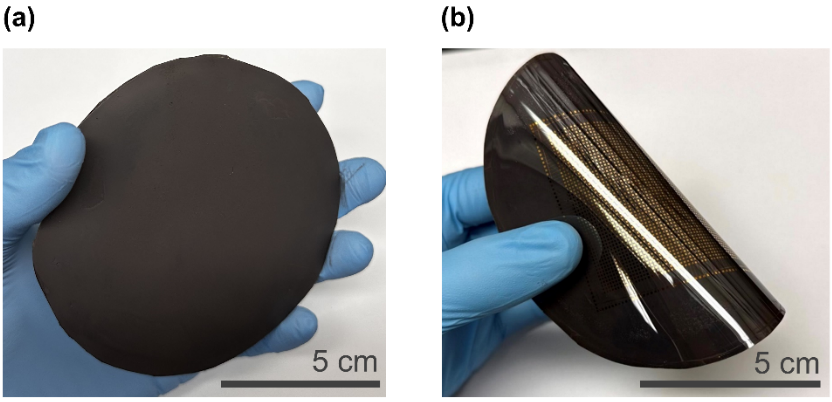

Supplementary Figure 2. Coating of BP ink film on 10 cm-diameter plastic film. (a) A BP ink film was coated onto a transparent PI/Au/PEDOT:PSS substrate, with the PI substrate held in place on the applicator using vacuum. For visual presentation, the coated PI substrate was cut into a circle with a radius of 5 cm. (b) The flexed polyimide substrate after detachment from the quartz, showing the bottom Au pads patterned as pixels of the imager. This imager demonstrates a scalable wafer-scale process for forming the BP film; however, the imaging results presented in the Article were not obtained using this imager.

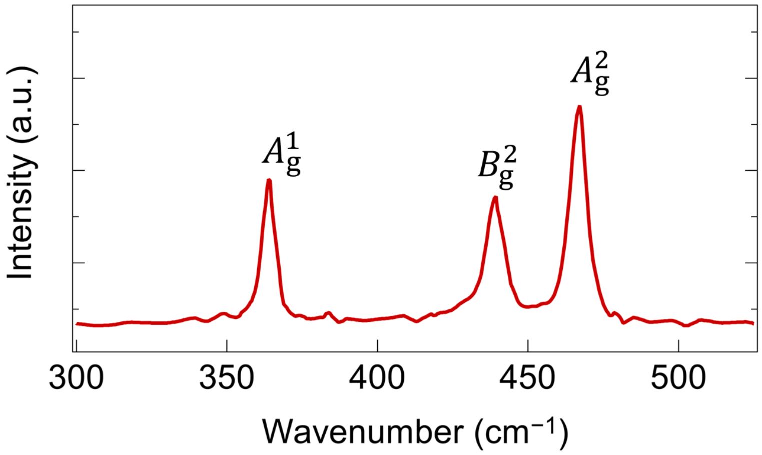

Supplementary Figure 3. Raman spectrum of the BP ink.

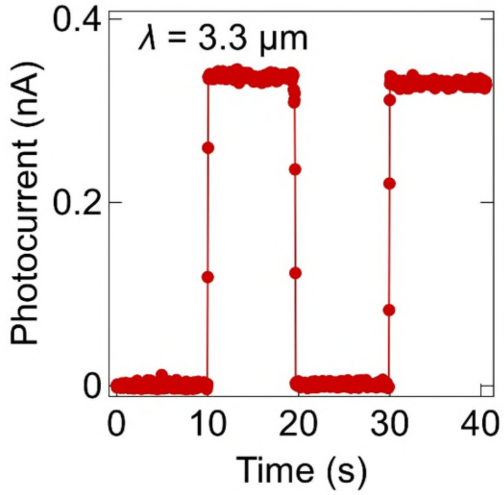

Supplementary Figure 4. MWIR photoresponse of the BP PD under irradiation from a λ = 3.3 μm LED (33 mW·cm^{-2}).

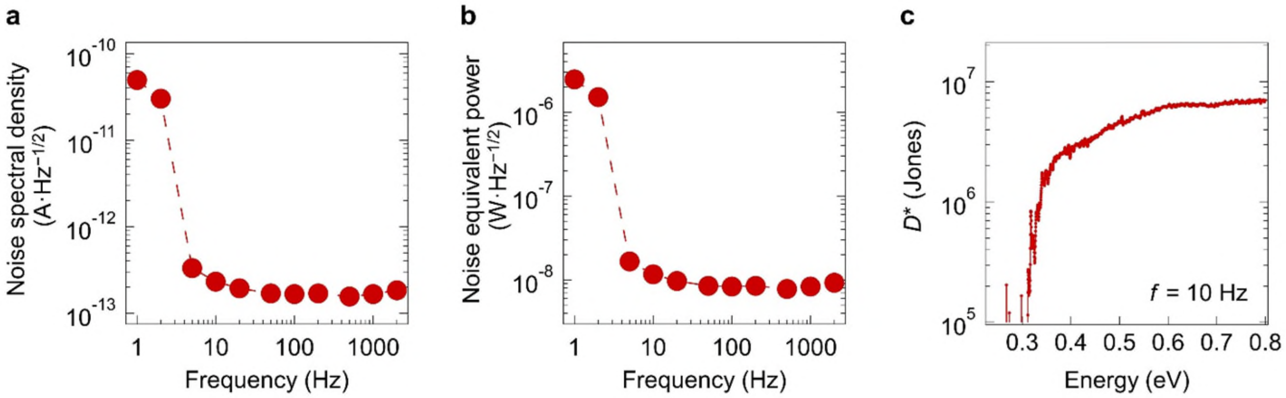

Supplementary Figure 5. Noise characterization and specific detectivity D^* analysis. (a) Noise spectral density (NSD). (b) Noise equivalent power (NEP) evaluated at λ = 3 μm. (c) Specific detectivity D^* as a function of photon energy at 10 Hz.

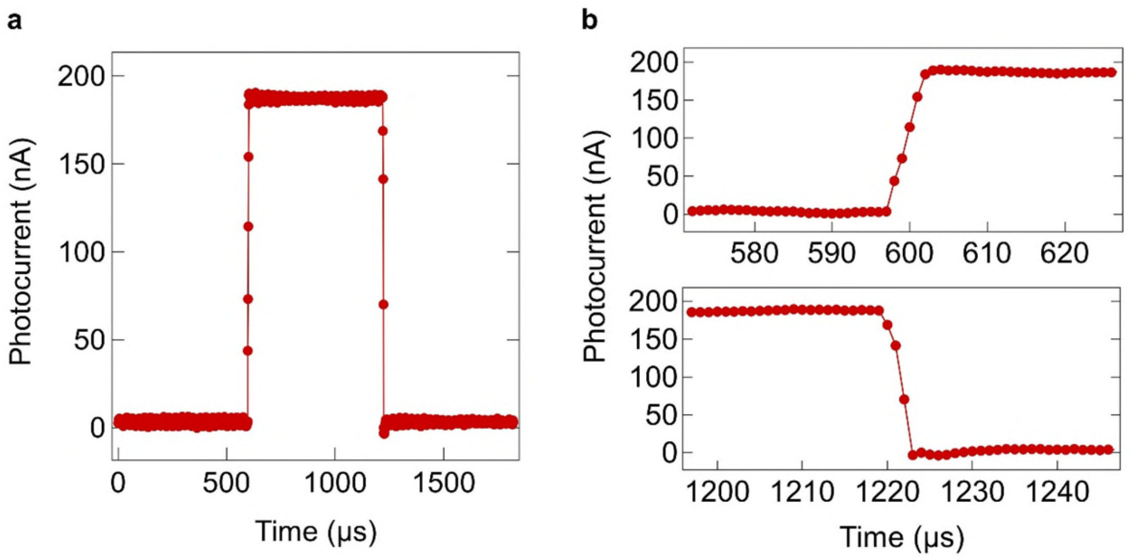

Supplementary Figure 6. Transient rise and fall behavior of the unbiased BP photodetector under λ = 1.55 μm laser irradiation (1.6 W·cm^{-2}). (a) Full measurement corresponding to Figure 2e. (b) Expanded view of the rising (top) and falling (bottom) segments, demonstrating the absence of long-tail slow components.

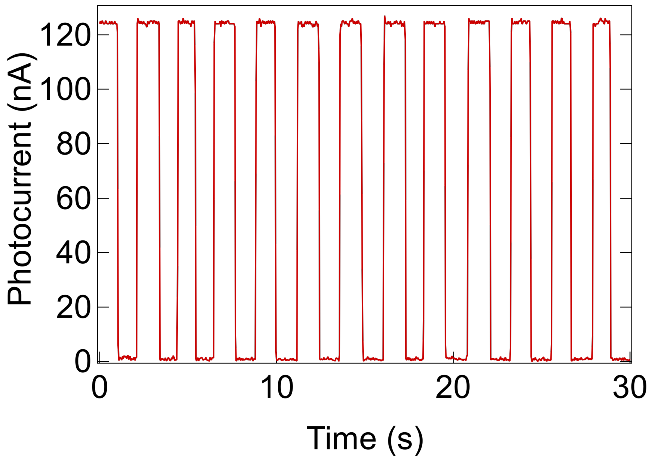

Supplementary Figure 7. Repeated switching cycles of an unbiased BP photodetector under λ = 1.55 μm laser irradiation (1.6 W·cm^{-2}), confirming reproducibility of the photoresponse.

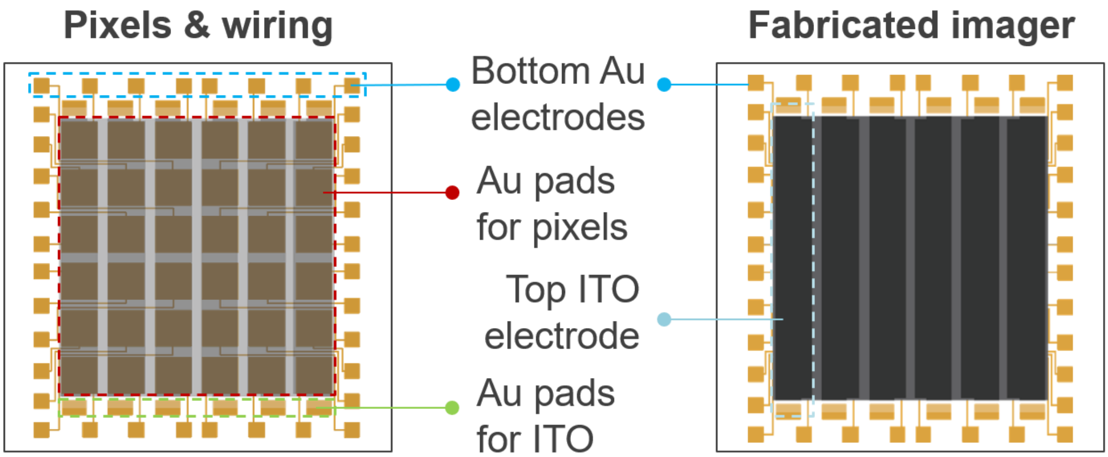

Supplementary Figure 8. Design of the imager. (Left) Schematic illustration of the pixels and wiring of the imager array, drawn to scale. The BP film and other functional layers are shown translucently to clearly reveal the bottom Au pads, which define the pixel area (750 µm × 750 µm). The Au pads adjacent to the top and bottom pixels of each row serve as readout pads for the partially overlapping top ITO electrodes. (Right) Schematic illustration of the fabricated imager, drawn to scale. The patterned BP film (gray) and ITO electrodes on the BP film (dark gray) are visible.

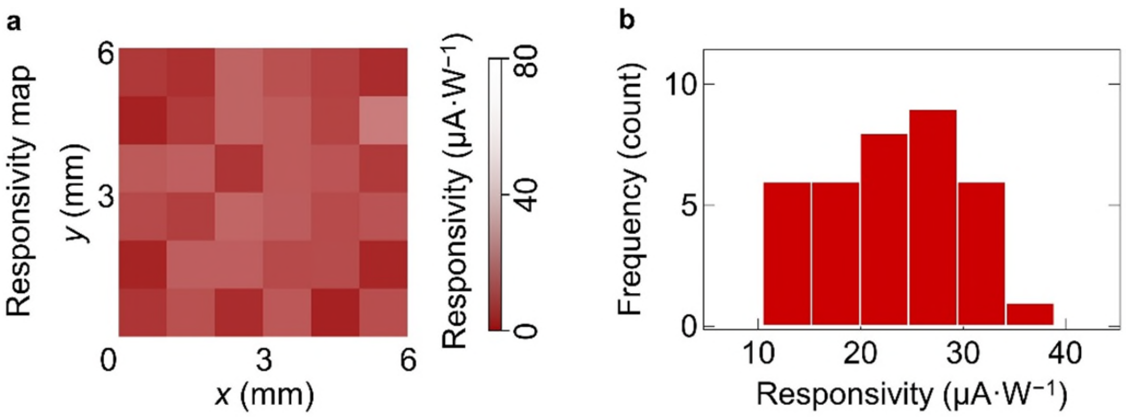

Supplementary Figure 9. Variation analysis of responsivity of the imager. (a) Spatial mapping of responsivity at 0 V under λ = 1.55 μm irradiation (1.6 W·cm^{-2}), measured across 36 pixels. (b) Histogram of the extracted responsivity values.

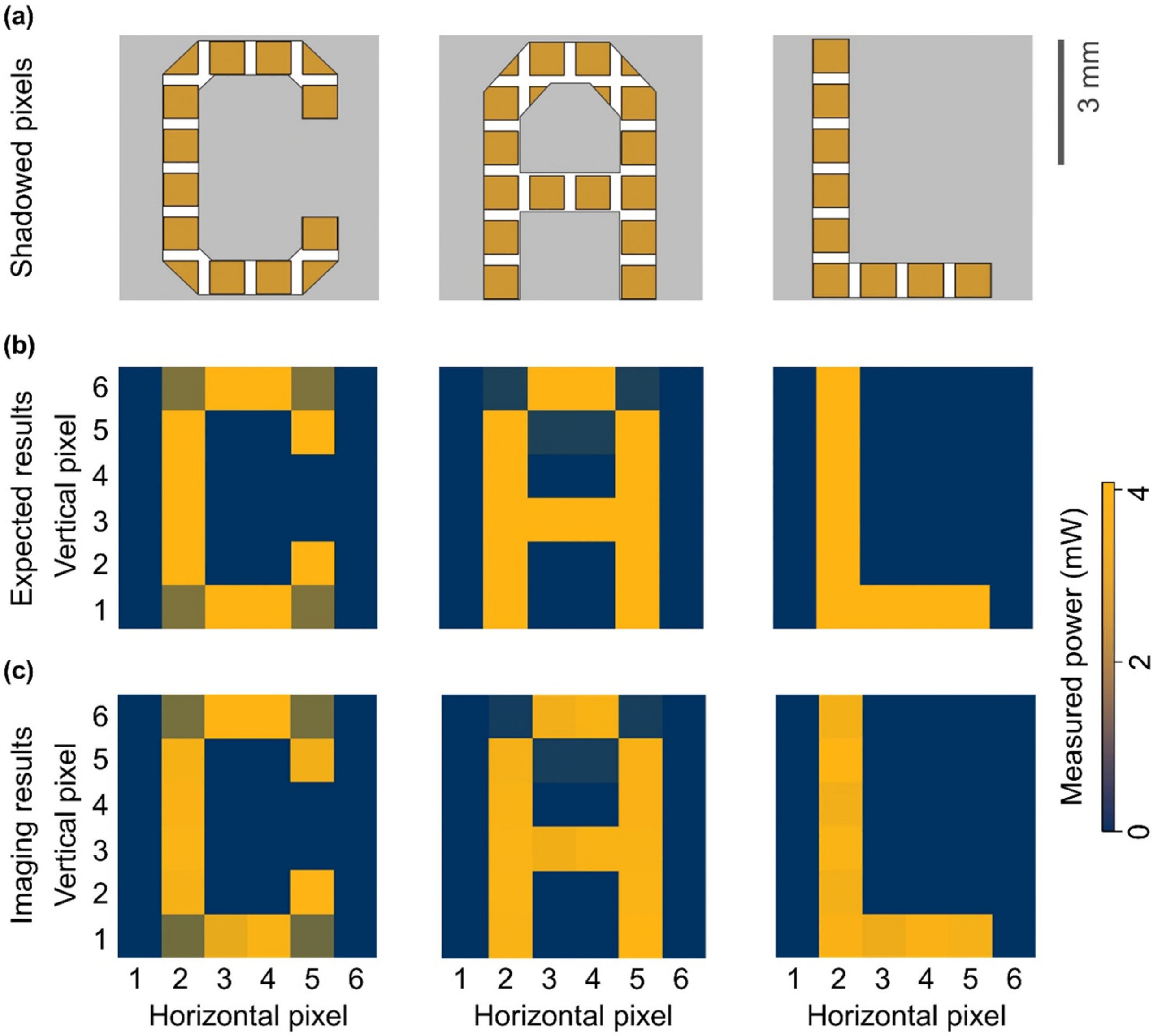

Supplementary Figure 10. Imaging of the letters “C”, “A”, and “L”. (a) Scaled illustration of the pixels with different shadow masks placed on top. (b) Simulated imaging results based on the calculated pixel areas exposed to the irradiating laser. (c) Experimental imaging results shown for comparison.

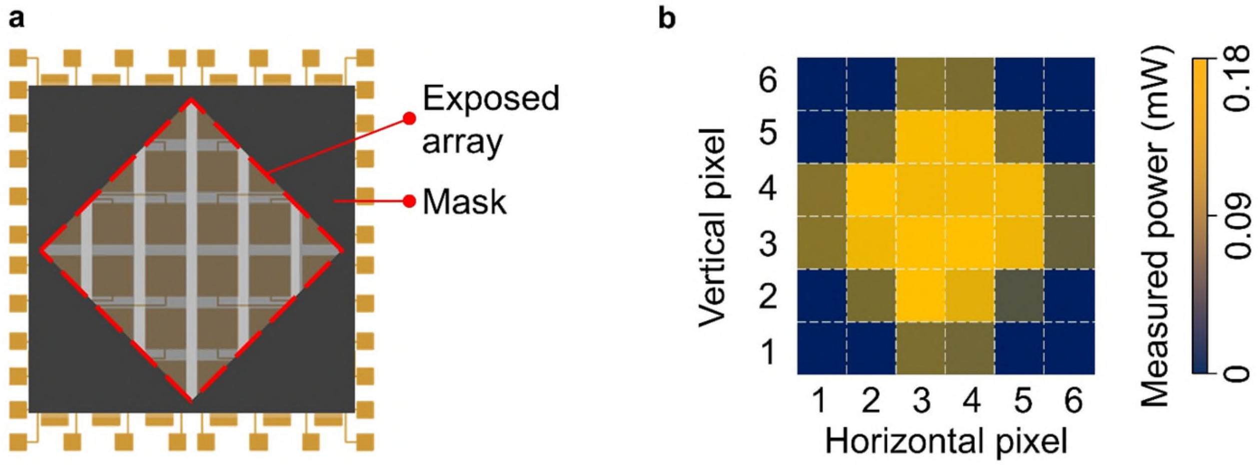

Supplementary Figure 11. Imaging using λ = 3.3-μm LED (33 mW·cm^{-2}). (a) Schematic configuration of the imaging pixel array and the shadow mask for MWIR imaging, showing a diamond-shaped exposed region. (b) Captured image revealing a recognizable diamond pattern. The photocurrent of each pixel is normalized to its maximum photocurrent.

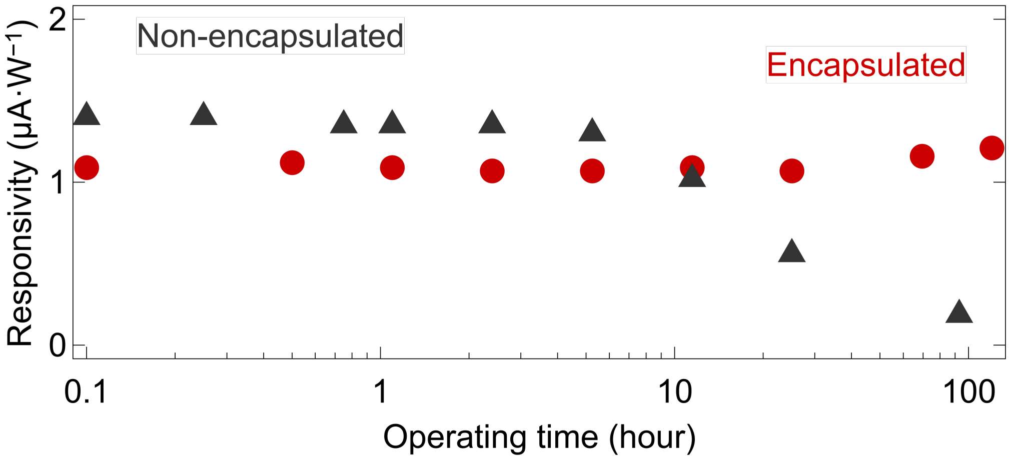

Supplementary Figure 12. Responsivity during the operational stability test of the encapsulated and non-encapsulated photodetector. Devices were illuminated by a 400 °C blackbody source (0.19 W·cm^{-2}) positioned 2 cm from the device.

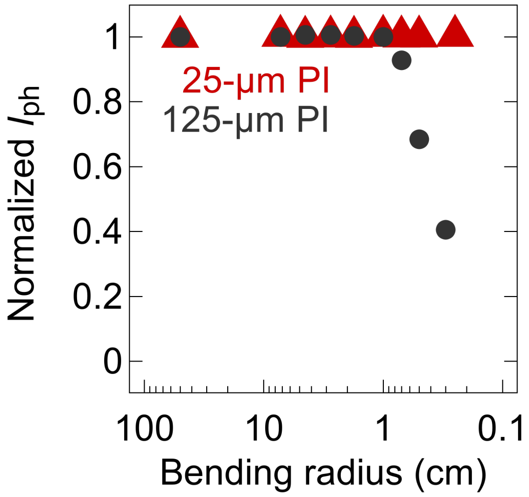

Supplementary Figure 13. Bending test of the BP photodetectors fabricated on PI films (thickness: 25 μm and 125 μm). The photocurrents are normalized to their respective initial values.

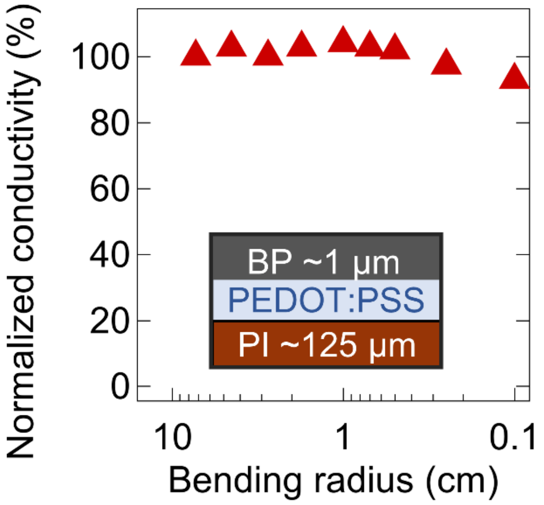

Supplementary Figure 14. Bending test of BP film coated on PI (125 μm)/PEDOT:PSS. Conductivity values are normalized to the initial measurement. (Inset) Schematic illustration of cross-sectional structure of the BP film. Electrical contacts on the BP film were formed using graphite-doped paste.

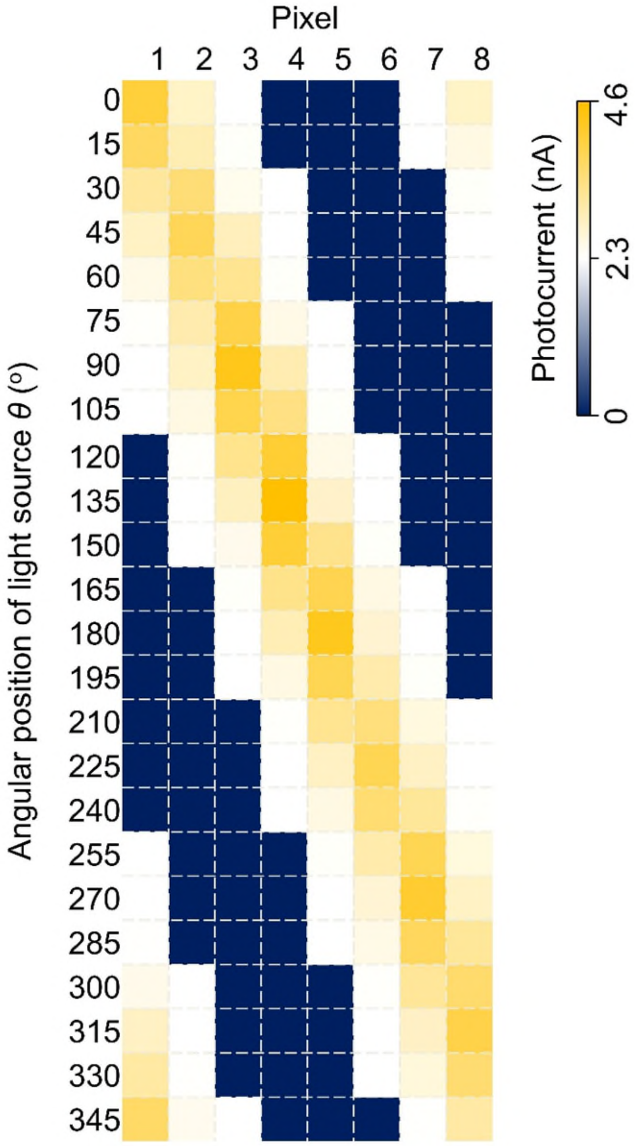

Supplementary Figure 15. Spatial mapping of photocurrent extracted from 1×8 flexible imager. Each row corresponds to the measured eight values for a given angular position of the light source (2796 K tungsten halogen lamp, 0.23 W·cm^{-2}).

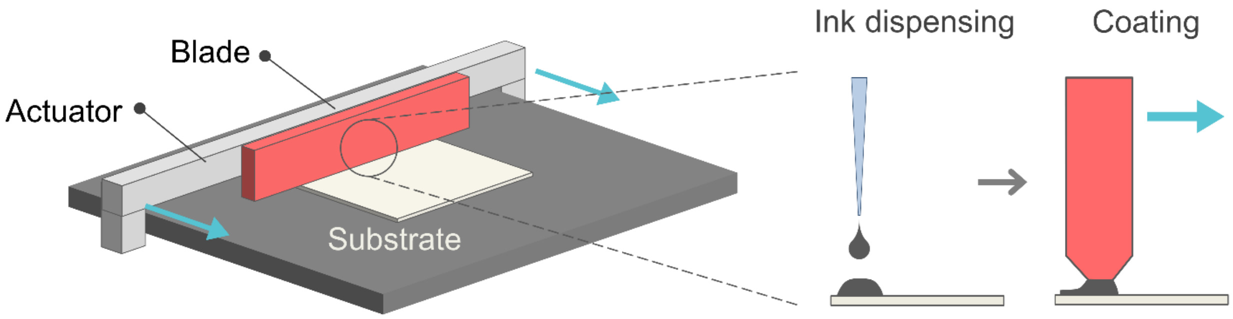

Supplementary Figure 16. Schematic illustration of the applicator. In this illustration, the actuator controls the movement of the blade to spread the dispensed ink. The blade height is adjusted to ensure precise contact with the ink on the substrate. The film forms as a result of the blade movement and the surface tension of the ink.

Supplementary References

1 Zhang, D. *et al.* Electrically functionalized body surface for deep-tissue bioelectrical recording. *arXiv preprint arXiv:2412.03749* (2024).

2 Gupta, N. *et al.* Large-scale efficient mid-wave infrared optoelectronics based on black phosphorus ink. *Science Advances* **9**, eadi9384, doi:doi:10.1126/sciadv.adi9384 (2023).

3 Corletto, A. *et al.* Scalable Fabrication of Black Phosphorous Films for Infrared Photodetector Arrays. *Advanced Science* **11**, 2403182, doi:https://doi.org/10.1002/advs.202403182 (2024).

4 Akhavan, S. *et al.* Graphene-black phosphorus printed photodetectors. *2D Materials* **10**, 035015 (2023).

5 Hu, G. *et al.* Black phosphorus ink formulation for inkjet printing of optoelectronics and photonics. *Nature Communications* **8**, 278, doi:10.1038/s41467-017-00358-1 (2017).

6 Mu, G. *et al.* Visible to mid-wave infrared PbS/HgTe colloidal quantum dot imagers. *Nature Photonics* **18**, 1147-1154, doi:10.1038/s41566-024-01492-1 (2024).

7 Xu, T. *et al.* Van der Waals mid-wavelength infrared detector linear array for room temperature passive imaging. *Science Advances* **10**, eadn0560, doi:doi:10.1126/sciadv.adn0560 (2024).
